# Supplementary material for: Specific Activation of Yamanaka Factors via HSF1 Signaling in the Early Stage of Zebrafish Optic Nerve Regeneration
Source: Int J Mol Sci. 2023 Feb 7;24(4):3253. doi: 10.3390/ijms24043253 (PMC9961437; doi:10.3390/ijms24043253)
Supplement: Supplementary file 1 [file ijms-24-03253-s001.zip › ijms-2163107-supplementary.pdf]

Figure S1

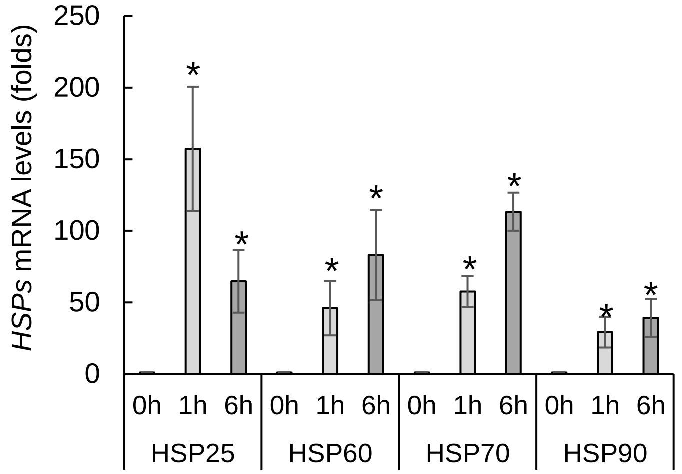

Figure S1. Upregulation of HSPs (HSP25, HSP60, HSP70, HSP90) mRNA in zebrafish retina after optic nerve injury. Data are expressed as the mean ± SEM of five to six independent experiments and analyzed by one-way ANOVA, followed by Scheffe's multiple comparisons. Statistical significance was set at $^*p < 0.05$.

Figure S2

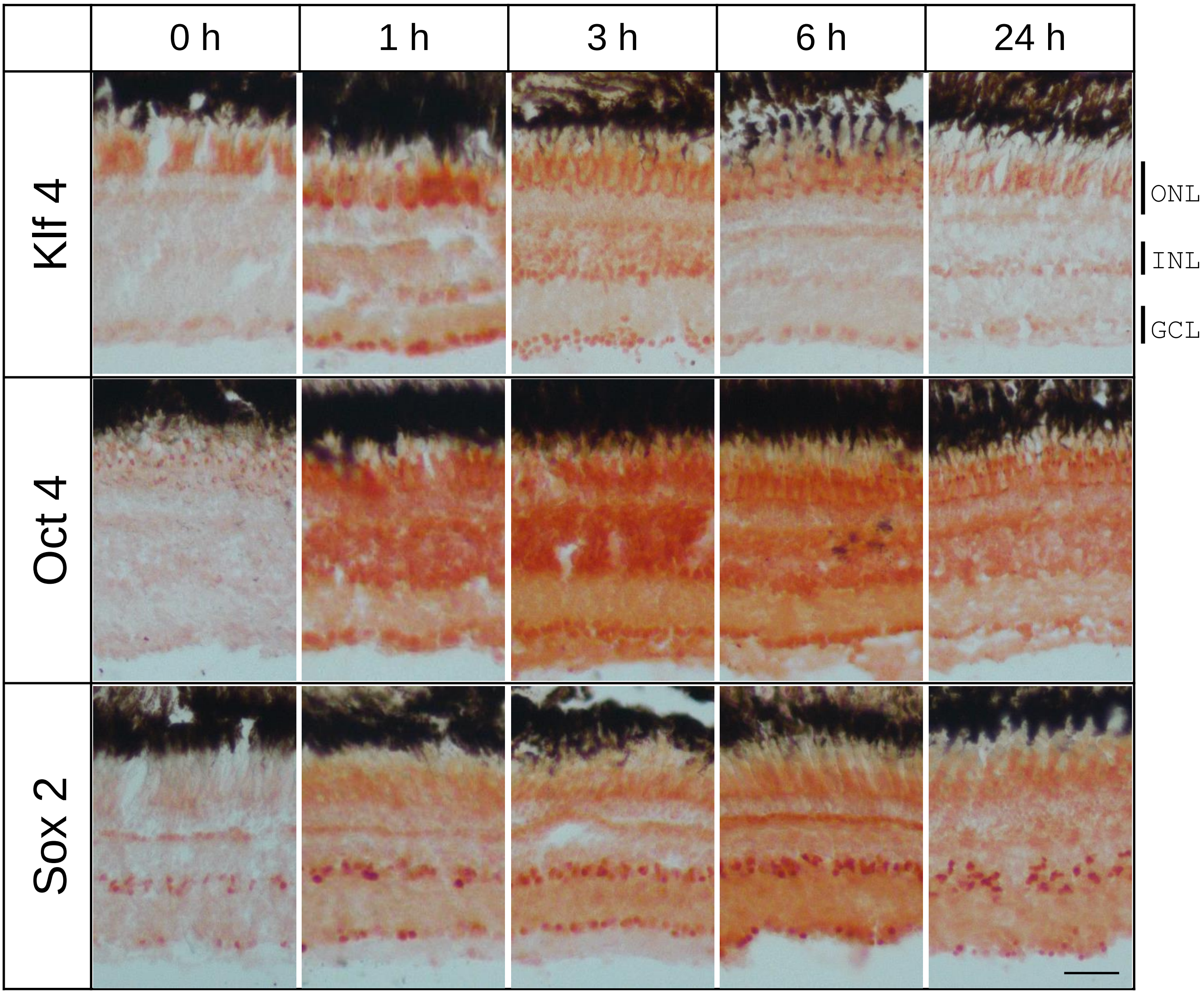

(Bar, 50µm)

Figure S2. Immunohistochemical staining of Klf4, Oct4, and Sox2 in the zebrafish retina after ONI. (Klf4) Significant immunostaining peaked at 1 to 3 hours in the ONL, INL, and GCL after ONI. (Oct4) Positive staining is detected in all nuclear layers 1-6 hours after ONI. (Sox2) Positive immunostaining for 1 to 24 hours in the ONL, INL, and GCL peaked at 6 hours after ONI.

Figure S3

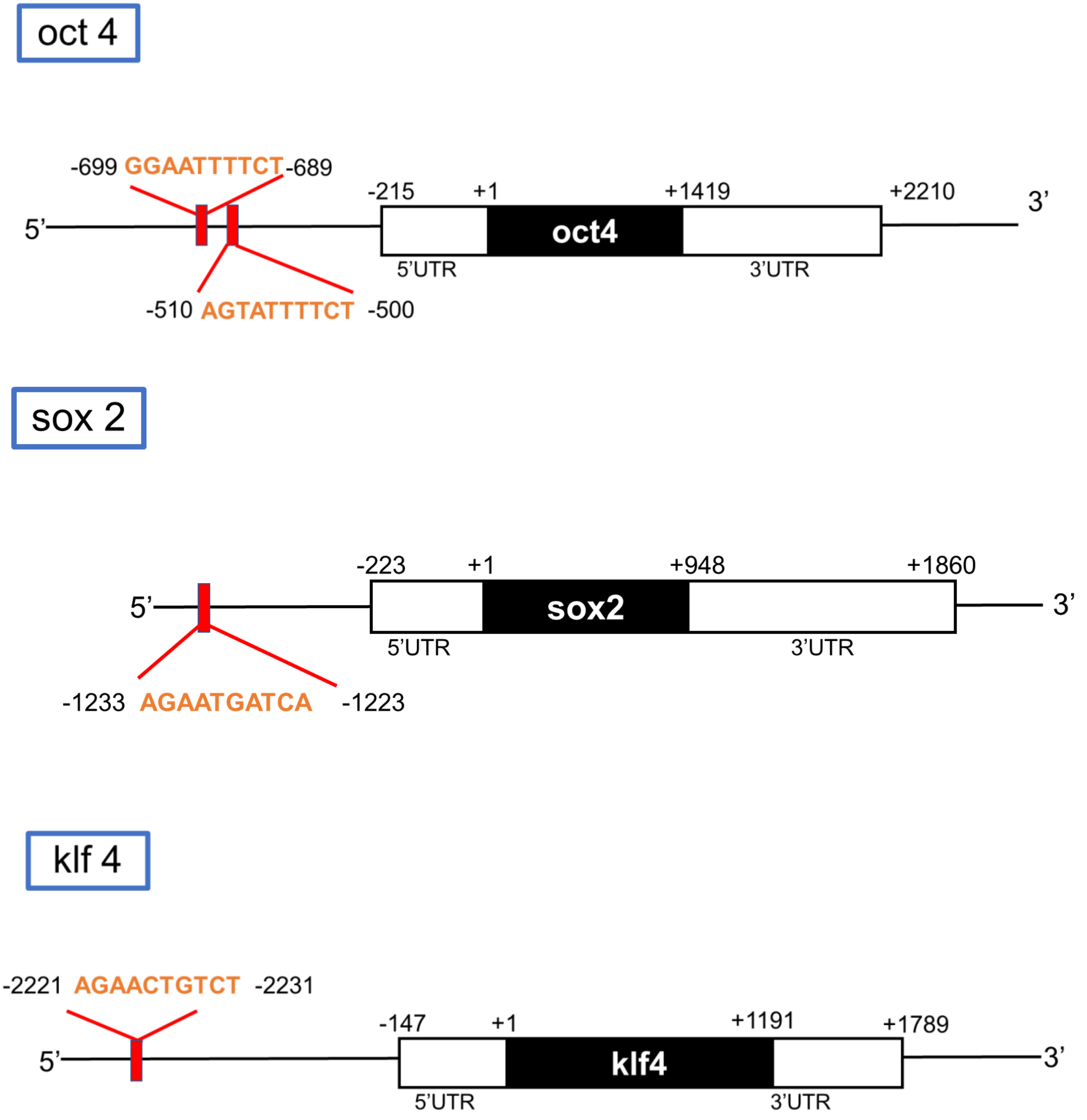

Figure S3.
Zebrafish OSK gene has a putative HSF1 binding site in their 5' promoter region. Each red bar indicates a putative HSF1 binding region by LASAGNA-Search 2.0.

Figure S4

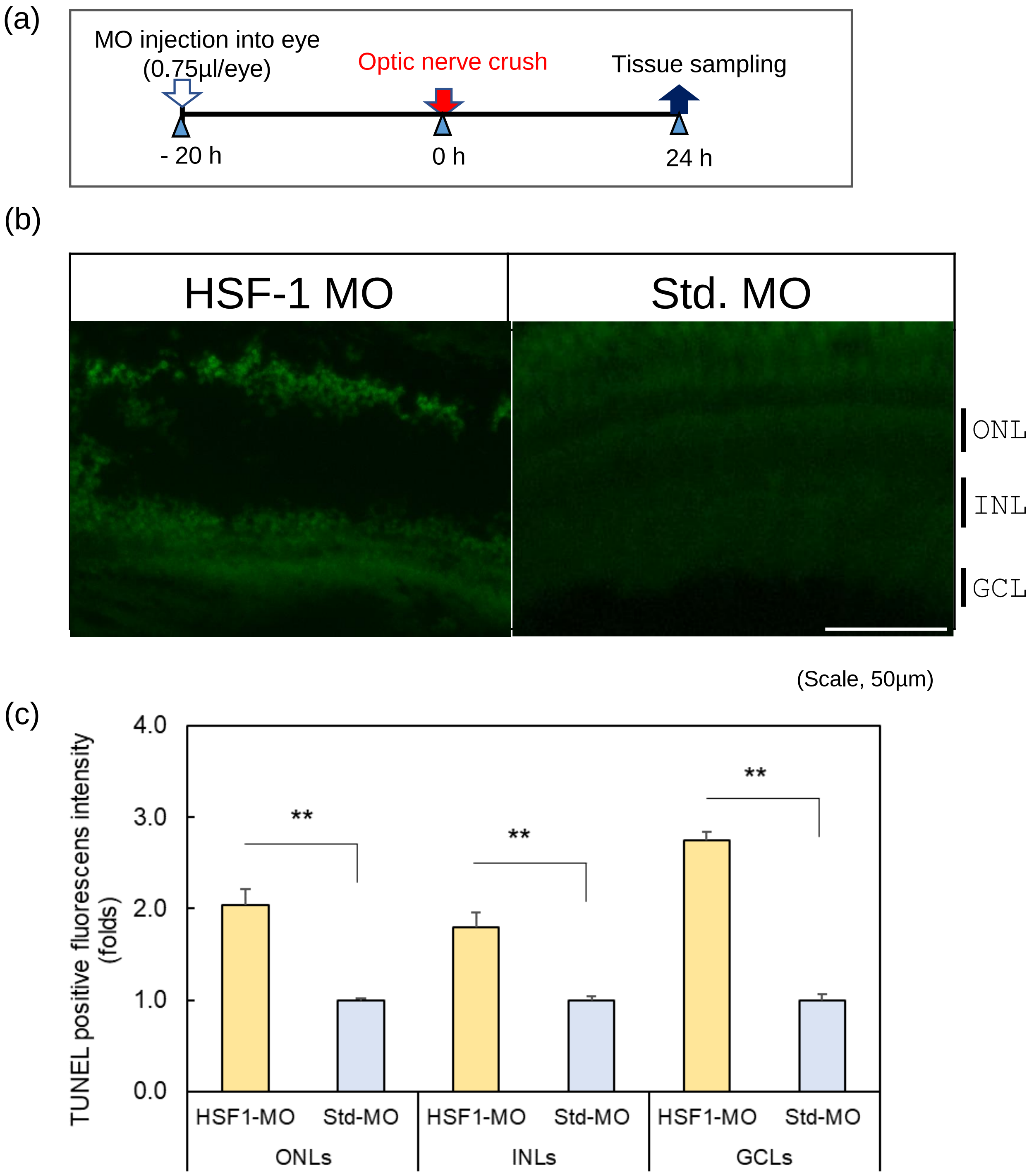

Figure S4. Detection of apoptotic cells after ONI under HSF1 knockdown conditions using the TUNEL (TdT-mediated dUTP nick end labeling) method. (a) Schedule of experimental procedures. HSF-1 MO or standard MO (Std. MO) was injected intraocularly 20 hours before ONI. (b) In the HSF-1 MO injection group, a large number of FITC-labeled apoptotic cells were observed in all nuclear layers, and the retinal layered structure was severely damaged. In contrast, no such phenomenon was observed in the Std MO group. (c) Apoptotic cell expression quantified by analysis of fluorescence intensity versus Std-MO control. Data are expressed as the mean ± SEM of five independent experiments and analyzed by one-way ANOVA, followed by Scheffe's multiple comparisons. Statistical significance was set at $^{**}p < 0.01$. Scale bar = 50 µm.
ONLs, outer nuclear layers; INLs, inner nuclear layers; GCLs, ganglion cell layers.

Table S1

Gene, mRNA accession number, primer name, product length, purpose, and primer sequences are shown.

Gene	Accession No.	Primer sequence	Amplicon	Probe	Purpose
HSF-1	AAI34899	Fwd, 5’-GATCTGCTGGAGCCCAAA-3’<br>Rev, 5’-TCGGCAGAACTTCTTTGGAA-3’	74 nt	#37	Real time PCR
		Fwd, 5’-AGTGGATTGAAGACCGAACG－3’<br>Rev, 5’-TGCTTGCCTGAATCAGTGTC-3’	475 nt	—	*In situ* hybridization
klf4	NM_001113483	Fwd, 5’-ACCGATGTGAAGCACAAGG-3’<br>Rev, 5’-GCAGGTCGCACCTGTAGAC-3’	108 nt	#12	Real time PCR
		Fwd, 5’-TTGATAGCATGGCACTGAGC-3’<br>Rev, 5’-GTGGAGGCTTGTAGGTTGGA-3’	476 nt	–	*In situ* hybridization
sox2	NM_213118	Fwd, 5’-GACCATTCATCGACGAAGCC-3’<br>Rev, 5’-CCTCCGGGGTCTGTATTTGT-3’	80 nt	—	Real time PCR
		Fwd, 5’-GGAACGGTAGGAACTCCACA-3’<br>Rev, 5’-TTTCATGTCAGCCTTTGCAG-3’	330nt	–	*In situ* hybridization
oct4	NM_131112	Fwd, 5’-CAACTCCCTCCGCTTCATC-3’<br>Rev, 5’-GCTTCCGAACCCATTTCC-3’	62 nt	#94	Real time PCR
		Fwd, 5’-GCTTAAACACAAGCGCATCA-3’<br>Rev, 5’-CGCTTTCCCTTCTGTCTACG-3’	411nt	–	*In situ* hybridization
